# Tympanostomy tube sequelae in children with otitis media with effusion: a three-year follow-up study

**DOI:** 10.1016/S1808-8694(15)31192-7

**Published:** 2015-10-20

**Authors:** Maria Beatriz Rotta Pereira, Denise Rotta Ruttkay Pereira, Sady Selaimen da Costa

**Affiliations:** 1 Otorhinolaryngologist, Master in Pediatrics, Federal University of Rio Grande do Sul, Coordinator of the Ambulatory of Pediatric Otorhinolaryngology, Sistema de Saúde Mãe de Deus (Porto Alegre), Fellow in Pediatric Otorhinolaryngology, University of Manitoba, Winnipeg, Canada.; 2 Medical studies under course, Medical School, Pontifícia Universidade Católica do Rio Grande do Sul.; 3 Assistant Professor, Ph.D., Department of Otorhinolaryngology and Ophthalmology, Medical School, Federal University of Rio Grande do Sul, and Coordinator of Ambulatory of Otology, Sistema de Saúde Mãe de Deus (Porto Alegre). Medical School, Federal University of Rio Grande do Sul.

**Keywords:** otitis media, otitis media with effusion, otitis media with effusion/surgery, ear, middle/surgery, middle ear ventilation, child

## Abstract

Tympanostomy tube (TT) insertion is one of the most frequently performed procedures in otolaryngology. Otorrhea, tympanosclerosis, retraction, perforation, and cholesteatoma are complications reported in the literature after its application. **Aim**: To determine the incidence and the type of TT insertion sequelae/complications in children presenting with recurrent otitis media and chronic otitis media with effusion undergoing myringotomy and tube placement. **Study Design**: prospective cohort study. **Material and Method**: A total of 75 children (150 ears) aged 11 months to 10 years were regularly followed up for up to 38 months after TT insertion. **Results**: Incidence of sequelae/complications: otorrhea - 47.3% of the ears; perforation - 2.1%; retractions - 39.7%; tympanosclerosis - 23.3%. Average length of stay: 12.13 months. Mean age at initial tube placement of children not requiring a second set of tubes = 35.9 months and mean age at initial tube insertion of children requiring an additional set of tubes = 25.6 months (P=0.04). TT stayed longer in the ears that had more episodes of otorrhea (P=0.01). TT insertion with adenoidectomy was associated with a smaller number of otorrhea episodes (P=0.02) **Conclusions**: Otorrhea was the most frequently found complication. TT placement with adenoidectomy was associated with fewer otorrhea episodes. TT extruded later in those ears that had more episodes of otorrhea. Younger age at the time of the initial tube placement is associated with higher incidence of additional tube placement. One in six patients will probably require a second set of ventilation tubes.

## INTRODUCTION

Since the reintroduction of ventilation tubes (VT) by Armstrong in 1954, myringotomy with VT insertion has been established as an effective treatment approach for otitis media with effusion (OME)^1^. OME is a middle ear inflammation in which there is retro-tympanic liquid collection, without signs or symptoms of acute infection and integral tympanic membrane ^2^. It is normally considered a direct continuation of the inflammatory process that takes place during prolonged and recurrent episodes of acute otitis media (AOM), which is confirmed not only by that fact that all cases of OME follow an episode of AOM, but also because of experimental studies in animals ^3^. There are approximately 2.2 million episodes of OME diagnosed every year in the United States, causing expenses of nearly US$4 billion ^4^. Currently, myringotomy with insertion of ventilation tubes is one of the two most common surgical procedures in North-American children and the main reason for children to take general anesthesia ^5^. Even though considered a simple procedure with significant benefits, VT insertion may present undesirable consequences. Tympanic membrane and middle ear sequelae in children with OME treated with VT have been reported many times in the literature. In many occasions, it is difficult to separate sequelae resulting from the disease from those resultant from treatment. Complications in insertion of VT include otorrhea, tympanosclerosis, tympanic membrane perforation, retractions and cholesteatomas 6, 7.

The identification of previously non-existing changes in the tympanic membrane (incidence) requires a prospective study with regular and appropriate follow-up. Differently from prevalence, data of incidence allow risk estimates for the development of sequelae during a specific period of time in those subjects that did not have it in the onset of the study.

Thus, we tried to determine types and incidences of sequelae/complications related with ventilation tube in a cohort of children with OME (children with recurrent otitis media and otitis media with chronic effusion), submitted to myringotomy and VT insertion and regularly followed up.

## MATERIAL AND METHOD

We conducted a cohort longitudinal prospective study with sub-individual data (ears).

Seventy-five children with diagnosis of OME, coming from the pediatric clinic of Otorhinolaryngology, Porto Alegre, were enrolled in the study during the period of June 2001 to October 2002, and they were followed up until July 2004. It included patients that were aged 11 months to 10 years, who had had middle ear effusion for six weeks or more (OME), with diagnosis of recurrent otitis media - ROM (three or more episodes of AOM within six months) or chronic otitis media with effusion - COME (persistence of effusion for over three months) that presented indication of myringotomy and ventilation tube insertion. The first author (MBRP) has performed the clinical follow-up of all selected cases for a minimum period of six weeks before the surgery. Immittanciometry was made, when necessary, to confirm the presence of effusion in cases of ROM and all patients with diagnosis of COME were assessed with audiometry and immittanciometry. We performed pneumatic otoscopy under video-endoscopic vision, in all patients, 24 hours before the surgical procedure to confirm the absence of signs and symptoms of acute infection. The patients that presented, at the time of the surgery, AOM, upper airway infections, current use of antibiotics or history of conclusion of treatment less than 7 days before, were excluded.

The surgical act was performed under general anesthesia. Middle ear effusion was aspirated with an Alden-Senturia collector (*Alden-Senturia collector*^®^, Storz Instruments, St. Louis, USA), because it had been included in a study about the presence of bacteria in middle ear effusion of children with OME ^8^. Short-term ventilation tubes, made of silicone, measuring 1.2 x 2.6 mm, type Donaldson (*Medicone*^®^, Pomp Produtos Hospitalares, Cachoeirinha, Brazil) were placed on the anterior-inferior quadrant of the TM.

Patients were examined with videoendoscopy in the following intervals: 7 and 30 days after surgery and then within 45-60 days or at any time if there were any complications. We assessed the time VT remained in place, frequency of otorrhea, perforations and other structural TM changes after extrusion of VT and number of episodes of AOM and OME after the extrusion of the VT.

Quantitative variables (age, duration of follow-up, and stay of VT) were described as mean (± standard deviation) and qualitative variables (surgical indication, type of surgery, occurrence of otorrhea, tympanosclerosis, retraction, perforation, AOM and OME after extrusion of VT), were presented by absolute and percentage frequency.

The analysis of data was conducted by Kolmogorov-Smirnov test (K-S). Out of the total quantitative variables, only VT time of stay had to be submitted to logarithmic transformation to be assessed by analysis of variance, considering its asymmetry. The comparison of means was carried out with analysis of variance (ANOVA) and comparisons of proportion of chi-square. The level of significance adopted was = 0.05.

Research data were stored in a database using the software MS Excel^®^, version 2002 for Windows XP. The statistical package we employed was Statistical Package for Social Sciences (SPSS^®^), version 11.0^9^.

The research protocol was approved by the Research Ethics Committee, Research and Post-Graduation Group at Hospital de Clínicas de Porto Alegre. Written post-informed consent was collected from all parents or guardians. The indication of the surgical procedure was made before patients were enrolled in the study, which was a pre-requirement for the inclusion in the sample.

## RESULTS

All 75 children included in the sample received insertion of VT bilaterally, totaling 150 operated ears. Ages ranged from 11 months to 9 years and 4 months (mean ± standard deviation = 34.7±18.5 months), 60% were boys and they were all Caucasians. Two patients dropped out the follow-up after the first postoperative assessment (one operated for ROM and the other for COME), which did not allow the planned monitoring. Thus, we followed up 146 years and 73 children.

We diagnosed ROM in 69.3% and COME in 30.7% of the patients. The patients with ROM had mean of 5.3±1.4 episodes of otitis per semester and those with COME presented an average time with middle ear with effusion of 4.8±1.1months.

Family history of AOM was detected in 45.3% of the patients and attendance to day care center was recorded in 89.3% of the children included in the study.

The patients were followed up for up to 38 months after insertion of the VT with a mean of 23.16±8.46 months. The mean number of reviews was 17.04±9.72 per patient. The mean stay of VT was 12.13±6.06 months (Table 1).

**Table 1:** Descriptive results for a total of 146 ears.

Variables	Frequency	%
Otorrhea		
None	77	52.7
1 episode	46	31.5
2 or more episodes	23	15.8
Perforation	3	2.1
Retraction	58	39.7
Tympanosclerosis	34	23.3
Cholesteatoma	0	0
Stay of VT (in months)*	12.13 (± 6.06)	
Duration of follow-up (in months)*	23.16 (± 8.46)	
Number of reviews*	17.04 (± 9.72)	

VT: ventilation tubes

* Variables presented as mean (± standard deviation)

Tables 1 and 2 present descriptive results of frequency, percentage and mean ± standard deviation of the studied variables. The variable otorrhea is shown in both tables because the relevant literature describes the occurrence both by ears and by patients. Thus, 61.6% of the patients presented otorrhea at some time in the follow-up, and 31.5% of the ears presented one episode of otorrhea and 15.8% had two or more episodes.

**Table 2:** Descriptive results for 73 patients.

Variables	Frequency	%
Otorrhea	45	61.6
Otitis media with effusion after VT extrusion	19	26.0
Right	1	1.4
Left	2	2.7
Both	16	21.9
Acute otitis media after VT extrusion		
None	26	35.6
1 to 3 episodes	36	49.3
Over 3 episodes	11	15.1
Surgical reintervention		
Not performed	61	83.6
VT	3	4.1
VT+Ad	9	12.3

VT: ventilation tubes

VT + Ad: ventilation tubes + adenoidectomy

The mean age in the first insertion of VT in those patients that did not require surgical reintervention was 35.9±19.1 months, whereas mean age of those who needed a new insertion of VT was 25.6±12.5 months, characterizing a statistically significant difference (P=0.04).

We tried to identify a possible correlation between reasons for VT insertion (ROM or COME) and occurrence of perforation, retraction, tympanosclerosis, need for surgical reintervention and stay of VT, and we did not find any statistically significant difference.

Given that otorrhea is a frequent and recurrent finding in patients with VT, we crosschecked some variables and the results are presented in Table 3. We detected that there was a significant increase in permanence of VT in ears that had more episodes of otorrhea (P=0.01). As to type of initial surgery, children submitted to VT placement with adenoidectomy (VT+Ad) presented a significantly smaller number of otorrhea episodes (P=0.02).

**Table 3:** Comparison of some variables concerning occurrence of otorrhea by ear.

**Variables**	**None**	**Otorrhea 1 episode**	**2 or more episodes**	**P**
Stay of VT (in months)*	11.06 (± 5.44)	1235 (± 5.97)	15.26 (± 7.26)	0.01
Age (in months)*	35.6 (± 13.97)	34.91 (± 24.20)	28.57 (± 18.31)	0.26
Indication of surgery **				
ROM	55 (52.9%)	29 (27.9%)	20 (19.2%)	
COME	22 (52.4%)	17 (40.5%)	3 (7.1%)	0.11
Type of initial surgery **				
VT	27 (40.9%)	24 (36.4%)	15 (22.7%)	
VT + Ad	50 (62.5%)	22 (27.5%)	8 (10.0%)	0.02

VT: ventilation tubes; VT + Ad: ventilation tubes + adenoidectomy

ROM: recurrent otitis media; OMEC: chronic otitis media with effusion

* Variables presented as mean (± standard deviation); using ANOVA

** Variables presented as frequency (percentage); using chi-square test

## DISCUSSION

The comparison of results of the present study with other publications was limited by the different definitions of OME, ROM and COME, such as indications for VT placement.

Regular follow-up of children by the same investigator to best characterize the type of OME and to follow up the duration of effusion is a difficult task in most clinical and pediatric ambulatory settings. Thus, the study we conducted had the advantage of including some patients of the main author, who was the sole responsible for initial assessment and postoperative follow-up that included otoscopy under endoscopic view in each visit. Moreover, it justifies the excellent compliance with postsurgical follow-up, with only 2 patients out of 75 lost to follow-up.

The mean time of VT permanence (12, 13 months) was similar to that reported by Collete et al.^10^ (13.8 months) and Casselbrant et al.^11^ (12 months). Given that the tube we used is a short duration VT, of dual reel-like setup, with estimated permanence of 6-18 months, we understand that the results we found were as expected.

The confirmation of tympanic membrane perforation after extrusion of the tube in 2.1% of the ears is also in agreement with the 3% rate reported by Daly et al.^6^ and the 2.2% found in the meta-analysis performed by Kay and Rosenfeld^5^ in a specific study on TM perforation carried out by Golz et al.^12^ These data reinforce that the incidence of perforations is small when we use short-duration tubes, because the two last studies presented rates of perforation of 16.6% and 14.5% for long duration tubes, respectively. Despite the reference by Golz et al.^12^ of a statistically significant difference between incidence of perforations depending on the surgical indication (COME = 1.56%; ROM = 16.4%), this fact was not confirmed in our study.

The incidence of tympanosclerosis (23.3% of the ears) did not differ statistically from the 32% reported by Kay and Rosenfeld^5^, but it was smaller than the rates of 40%, 48% and 53% reported by Daly et al.^6^, Schilder et al.^13^ and Sederberg-Olsen et al.^14^

Cholesteatomas were not detected in the ears we followed up and it was probably due to the relatively short period of follow-up (average of 23.16 months) and low incidence of this complication was 0.7% in the meta-analysis carried out by Kay and Rosenfeld^5^, 0.6% in Giebink study ^15^ and estimated between 0.1 and 1% by Casselbrant and Mandel^16^.

The retraction rate of TM was 39.7% and it was reported as 26% by Maw and Bawden^17^, 28.1% by Kay and Rosenfeld^5^, 37% by Tos et al.^18^ and 47% found in the study of prolonged follow-up (7 years) by Daly et al.^18^, and we shall point out that we did not find retraction pouches. As mentioned before, the follow-up of children in this study was reduced when compared to some studies performed in other countries; it hindered the appropriate valuation of data referring to retraction, considering that some of them could have developed it later, as a consequence of auditory tube dysfunction. Moreover, lack of uniform definition for retraction among the reported authors is also a restrictive element, considering that the retraction pouches, retractions of *pars flacida* and *pars tensa* were also reported either together or separated.

It is known that AOM and OME episodes happen after extrusion of VT in some children. At the present study, a total of 26% of the children developed OME after the tube was extruded and 64.4% presented at least one episode of AOM. It is believed that the VT replaces the auditory tube function, but the normalization of the functioning of the tube may take years, and thus, new episodes of otitis media may be expected. Authors such as Muenker^19^ do not even consider them as complications. In our series, these episodes were clinically controlled in most of the times, but 16.4% of the children required a second intervention with VT, frequency similar to the 19.9% obtained in patients reoperated on by Boston et al.^20^ Children that were treated with a second pair of VT were significantly younger at age (25.6 months) when the first VT was inserted than those that did not require reintervention (35.9 months), which confirms the findings by Boston et al.^20^ who reported that the need to reinsert VT was almost twice higher in the group of younger children when they performed the first surgery. These data seem to agree with peak ages of AOM and OME that are at one and two years, respectively, reinforcing the role of immune maturation and the development of middle ear and craniofacial structures in the decrease of the rates of these diseases. Given that we still discuss whether early surgical intervention prevents anatomical sequelae and speech and language development delays, the risk of requiring a second VT insertion should be considered in children who have early indication for myringotomy + VT insertion.

Nine out of 12 children that needed VT reinsertion suffered also an adenoidectomy (the others had already been submitted to the procedure in the first surgical act). There are different opinions about the benefits of adenoidectomy in treating COME and ROM. No all mechanisms that justify the positive effect are well known. Paradise et al.^21^ recommended adenoidectomy in children who had ROM after extrusion of VT and Valtonen et al.^22^ performed adenoidectomy whenever they performed reinsertion of VT. Current guidelines of the American Academy of Pediatrics and American Academy of Otorhinolaryngology, Head and Neck Surgery ^23^ reported that eight adenoidectomies are required to prevent one single VT reinsertion, but it reminds that each one of the prevented reinsertions when performing adenoidectomy represents a reduction in the incidence of AOM and OME.

Otorrhea is one of the most common complications after VT insertion. In our series, 61.6% of the patients presented otorrhea at some point in the follow-up, and 31.5% of the ears presented one episode of otorrhea and 15.8% presented two or more episodes. An elegant study conducted by Collette et al.^10^ showed an incidence of otorrhea in 74.8% of the children whose VT remained in place between 12-18 months. The literature shows a wide variation in frequency of otorrhea: Kalcioglu et al.^7^ reported an extremely low rate (1.25% of the 239 children) and the meta-analysis performed by Kay and Rosenfeld^5^ pointed to a percentage of 42%.

In the present study, there was significant increase in permanence of VT in the ears that presented two or more episodes of otorrhea (P=0.01). It is in agreement with the concept of the tube that takes longer to be extruded in situations in which there are middle ear mucosa abnormalities. There was also a significant decrease in the number of otorrhea episodes when adenoidectomy was performed together with the first insertion of VT (P=0.02). It is likely that the benefit of adenoidectomy is related with removal of the potentially infected tissue focus in the nasopharynx, since that it is known that the bacterial population of the nasopharynx is similar to that found in the middle ear of children with OME^24^, ^25^.

## CONCLUSION

Otorrhea was the most frequent sequelae/complication of ventilation tube insertion in a cohort of children submitted to myringotomy and ventilation tube insertion by OME.

Children who were submitted to ventilation tube placement with associated adenoidectomy presented fewer episodes of otorrhea.

The ventilation tube remained longer in the ears that had higher frequency of otorrhea.

Younger age at the first VT insertion was associated with higher incidence of the VT reinsertion.

The analysis suggested that 1 in each 6 patients submitted to ventilation tube placement will require reinsertion of a second pair of tubes.

The authors emphasized the importance and need for appropriate and regular follow-up of children in whom the ventilation tubes were placed, even after their extrusion, because sequelae are common.
